# Identifying Optimal Processing Variables and Investigating Mechanisms of Grain Alignment in Hot-Deformed NdFeB Magnets through Design of Experiments

**DOI:** 10.3390/ma17133371

**Published:** 2024-07-08

**Authors:** Jongbin Ahn, Jung-Goo Lee, Wooyoung Lee

**Affiliations:** 1 Department of Materials Science and Engineering, Yonsei University, Seoul 03722, Republic of Korea; jongbin7645@yonsei.ac.kr; 2 Department of Magnetic Materials, Korea Institute of Materials Science, Changwon 51508, Republic of Korea

**Keywords:** hot-deformed NdFeB magnet, design of experiment (DoE), minimal stress deformation

## Abstract

This study introduces a novel approach for investigating hot-deformed NdFeB magnets by combining the minimal stress deformation process (MSDP) with the design of experiment (DoE) methodology. This study focused on enhancing the crystallographic alignment, particularly the c-axis alignment of the Nd_2_Fe_14_B grains, to optimize the magnetic properties. By utilizing the Box-Behnken design matrix and response surface regression, critical processes and variables were identified, determining that a hot-pressing temperature of 700 °C is crucial for achieving optimal grain alignment. Changing the strain rate to 0.019 mm/s under a stress of 110 MPa led to significant enhancements in the alignment, yielding magnets with a remanence of approximately 13.4 kG and a coercivity of 21 kOe. These findings highlight the effectiveness of combining the MSDP and DoE for predicting and achieving improved magnetic properties. Despite the challenges associated with understanding the complexity of crystal alignment mechanisms, this integrated approach successfully improved magnetic characteristics. The methodology represents a significant advancement in the fabrication of high-performance hot-deformed NdFeB magnets, marking a notable contribution to the field.

## 1. Introduction

The recent surge in the demand for NdFeB magnets across industries such as automotive, aerospace, and electronics is attributed to their high magnetic properties. In particular, their roles in electric vehicle traction motors cannot be overstated. These motors require a high remanence, large coercivity, and flexible magnet shapes for improved performance [1]. When manufacturing NdFeB using the sintering process, methods such as grain boundary diffusion (GBD) have been used to enhance the coercivity [2,3,4]. These methods present significant challenges. The manufacturing process is limited to producing magnets of specific sizes suitable for GBD [2]. Despite these limitations, high-performance magnets are produced and supplied to major industries by applying the GBD process to block-shaped magnets. In sintered NdFeB magnets, these methods cause a reduction in remanence, incur considerable costs, and can lead to supply issues owing to their reliance on heavy rare-earth elements [5,6,7]. Alternatives such as rare earth bonded magnets and ferrite magnets also have limitations, offering inferior magnetic properties [8,9].

The hot-deformation process of rapidly solidified NdFeB ribbons, originally developed by Lee et al. [10], has emerged as a promising alternative for producing anisotropic magnets with excellent magnetic properties and an ultrafine grain size of approximately 250 nm, without shape restrictions [11,12]. Unlike sintered NdFeB magnets, in which crystallographic alignment results from magnetic alignment, the alignment of hot-deformed NdFeB magnets results from crystallographic alignment [13]. This technique provides a substantial advantage for attaining high magnetic properties without the need for additional processes, such as cold isostatic pressing, rubber isostatic pressing, or GBD [5,14]. These enhancements are primarily due to the ultra-fine and well-aligned grain structure achieved through hot deformation, which offers increased design flexibility for creating magnets with intricate shapes and geometries. This makes the process particularly attractive for applications in electronics, aerospace, and innovative automotive concepts [1,15,16].

Therefore, recent studies have actively enhanced the properties of hot-deformed NdFeB magnets [17,18,19,20,21,22]. Mouri and Wang [23,24] achieved a high remanence exceeding 13 kG and a coercivity below 15 kOe. Furthermore, J. Li [25] utilized the Nd_60_Tb_20_Cu GBD process and achieved a high remanence of 1.4 T and a coercivity of 2.56 kOe in 2 mm thick hot-deformed NdFeB magnets, indicating a promising direction for the further enhancement of magnetic properties in such magnets. However, as illustrated here, while significant advancements have been made, the current levels of remanence and coercivity, along with the challenges in achieving the desired thickness, maintaining shape integrity, and ensuring scalability for mass production, render the application of these magnets in high-performance motors, such as traction motors, difficult. Nonetheless, the most effective strategy for enhancing magnetic properties involves optimizing the grain alignment. Improved grain alignment directly correlates with higher magnetization [26], making the manufacturing of hot-deformed magnets through optimized grain alignment an ideal approach. This methodology underscores the potential for tailored applications, particularly in the design of magnets that satisfy the stringent requirements of advanced motor technologies without compromising performance.

We propose a novel approach by integrating the design of experiment (DoE) framework to determine the optimal parameters and introducing the minimal stress deformation process (MSDP) for grain alignment in NdFeB hot-deformation research. The MSDP is an innovative deformation method that maintains a nominal stress below 100 MPa when the true strain is below 1.0 during high-temperature deformation. This approach specifically aims to induce deformation at the grain boundaries, thereby enhancing the grain alignment to achieve high remanence. This method was designed to enhance remanence while preserving high coercivity, distinguishing itself by effectively controlling the deformation processes at the grain boundaries to maximize the magnetic properties.

Our comprehensive experimental strategy involved hot pressing at 675–725 °C for a duration of 20 min. This was followed by hot deformation at 785–815 °C using the MSDP. The DoE framework was instrumental in determining the optimal parameters that yield the peak magnetic properties. These findings were validated through an extensive scale-up process. This study aims to pave the way for the next generation of NdFeB magnets, address and overcome the limitations of previous fabrication techniques, and establish a robust pathway for enhancing their magnetic properties in high-performance applications.

## 2. Materials and Methods

Melt-spun ribbons with a composition of Nd_4.32_Pr_5.55_Dy_0.78_Fe_80.16_Ga_0.52_Co_4.47_B_4.19_ at% (Magnequench Co., Ltd., Tianjin, China) were used in the investigation of NdFeB magnets. Prior to the hot pressing and hot deformation processes, we characterized the magnetic properties of the ribbons. The raw material powder displayed a remanence (Br) of approximately 7.32 kG and a coercivity (Hcj) of 22 kOe, providing a baseline for the further analysis and comparison of the post-processed magnetic properties. These ribbons underwent hot pressing in vacuum (10^−5^ Torr) at temperatures of 675–725 °C for 20 min, under a pressure of 70 MPa, yielding samples designated as HP675, HP700, and HP725. Subsequently, the hot-pressed samples were subjected to hot deformation in vacuum at 785–815 °C to achieve anisotropic platelet-like grains [10,13]. The resultant samples were referred to as HD785, HD800, and HD815. The MSDP was employed during the hot deformation process to promote the alignment of the Nd_2_Fe_14_B grains. Deformation rates between 0.019and, 0.039 mm/s, as per the DoE matrix, resulted in samples labeled DR0.019, DR0.029, and DR0.039. The extent of deformation was determined using the percentage height reduction and true strain measurements. The Box–Behnken design (BBD) based on the response surface methodology was applied to examine the effect of the hot-pressing temperature, hot-deforming temperature, and deformation rate on the hot deformation of the magnets.

The magnetic properties of the hot-deformed samples were measured using a Magnet Physik Permagraph C-300 BH loop tracer. The crystal orientation of the deformed magnets was observed using X-ray diffraction (XRD; Malvern Panalytical, Malvern, United Kingdom) with Cr-kα (λ = 2.28976 Å) radiation. The microstructures were investigated using field-emission scanning electron microscopy (FE-SEM; TESCAN mira3, Brno, Czech Republic). The elemental distribution and grain boundary phase were analyzed using scanning transmission electron microscopy (STEM; JEM-ARM300F, JEOL Ltd., Akishima, Tokyo, Japan) combined with energy-dispersive X-ray spectroscopy (EDS).

## 3. Results and Discussion

### 3.1. Microstructural Evolution and Deformation Behavior of Hot-Pressed NdFeB Magnets

**Figure 1:**
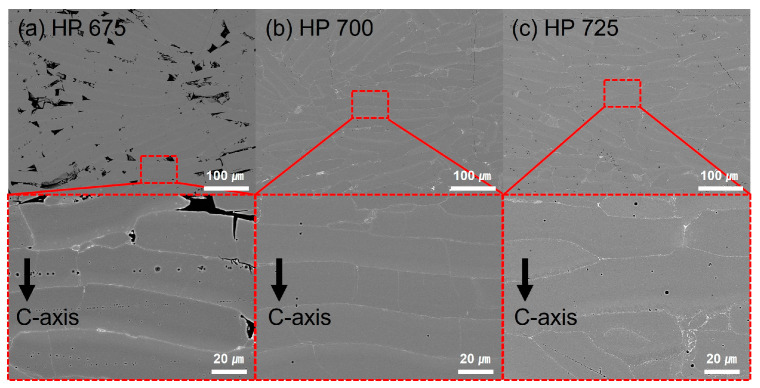
Backscattered electron-scanning electron microscope (BSE SEM) image of hot-pressed samples; (**a**) HP675, (**b**) HP700, and (**c**) HP725 samples.

The microstructural evolution and high-temperature deformation behavior of the hot-pressed NdFeB magnets were investigated at various hot-pressing temperatures. The melt-spun ribbons were subjected to hot pressing in vacuum at 675–725 °C for 20 min. Figure 1 illustrates the vertical cross-section microstructure of the samples hot-pressed at 675, 700, and 725 °C. The images show a melt-spun shape, characterized by a gray contrast separated by a bright contrast. As the hot-pressing temperature increased, the number of vacancies between the melt-spun flakes decreased, leading to an increased density in the hot-pressed samples. Table 1 lists the density, remanence, and coercivity values obtained at different hot-pressing temperatures. The remanence values increased from 6.94 to 7.93 kG as the hot-pressing temperature increased from 650 to 700 °C, stabilizing at approximately 7.93 kG between 700 and 750 °C. These findings align with the results reported by M. Lin [25] regarding density and remanence. Additionally, hot-pressing temperatures of 725 °C or higher induced grain coarsening at the melt-spun flake interface and the aggregation of rare-earth (RE)-rich phases. The presence of the RE-rich layer was influenced by the hot-pressing temperature [27,28]. Notably, densification was significantly enhanced by increasing the hot-pressing temperature, and the density of the HP700 magnet reached the theoretical value. Grain coarsening near the theoretical density is not expected to have a significant influence on the coercive and remanence properties of hot-pressed isotropic magnets.

**Figure 2:**
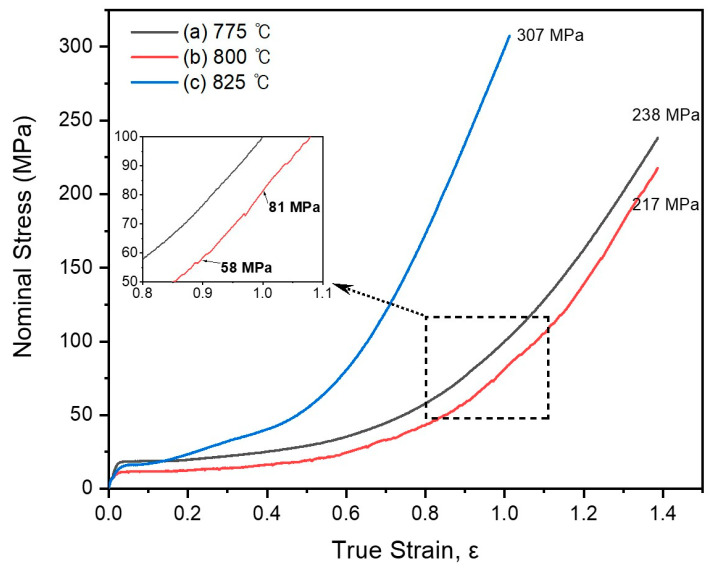
Nominal stress–true strain hot compression test at a deformation rate of 0.019 mm/s and compression test temperatures of (**a**) 775, (**b**) 800, and (**c**) 825 °C.

A compression test was conducted at 775, 800, and 825 °C to investigate the high-temperature plastic deformation behavior. The hot-pressed isotropic magnet (HP700) had a diameter of 20 mm and a height varying between 18 and 20 mm. Each compression test was conducted under atmospheric conditions, at a consistent strain rate of 0.02 mm/s. Figure 2 shows the nominal stress–true strain curves obtained from these tests. Figure 2a–c show the curves from the test at 775, 800, and 825 °C, respectively. In this study, the “nominal stress” was defined as the applied force divided by the original area of the specimen. Hereafter, we will use “stress” to refer to “nominal stress” for brevity.

During the test at 825 °C, a stress of 307 MPa was observed up to a true strain of approximately 1 ε. During the tests at 775 and 800 °C, stresses of 238 and 217 MPa, respectively, were observed up to a true strain of approximately 1.4 ε. Notably, during the test at 800 °C, the stress required for deformation to a height strain of approximately 60% (true strain between 0.9 ε and 1 ε) ranged between 58 and 81 MPa. Such findings indicate the potential of the HP700 magnet for high-temperature plastic deformation at 800 °C under a diminished applied pressure. The specimen exhibited successful plastic deformation, leading to the conceptualization of the MSDP. The temperature of 800 °C was highlighted as the ideal for achieving superior hot deformation and a pronounced c-axis texture. Above 800 °C, augmented stress due to grain growth and surface oxidation led to more evident crack formations, complicating the deformation process.

### 3.2. Crystallographic Alignment and Remanence Enhancement in Hot-Deformed Magnets: An XRD Analysis

**Figure 3:**
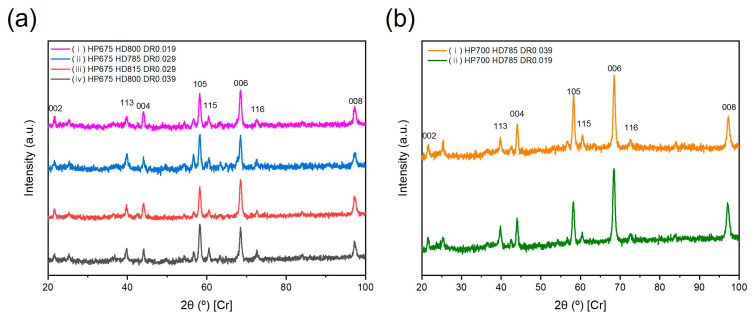
X-ray diffraction (XRD) patterns of hot deformed magnets procued by (**a**) HP675 + HD785~HD815 with DR0.019, DR0.029, and DR0.039 and (**b**) HP700 + HD785 with DR 0.019 and DR0.039.

Figure 3 shows the XRD patterns of the hot-deformed magnets at various temperatures (785, 800, 815 °C). The deformation rates were denoted as DR 0.019 (0.019 mm/s or 790 s), DR 0.029 (0.029 mm/s or 520 s), and DR 0.039 (0.039 mm/s or 390 s). The magnetic properties of the samples depicted in Figure 3 are detailed in Table 2. All the XRD patterns of the hot-deformed magnets, with remanence values between 12.0 and 13.4 kG, highlighted the anisotropic nature of the tetragonal structure of Nd_2_Fe_14_B along the c-axis.

Figure 3a illustrates the variations in the crystallographic alignment based on the deformation rate at a hot-pressing temperature of 675 °C. Figure 3b shows the alignment differences at a hot-pressing temperature of 700 °C. Figure 3a,b show (006), (105), (116), (115), and (113) reflections of varying intensities. In Figure 3a, the intensities of the (006) and (105) reflections are comparable. However, as shown in Figure 3b, the peak intensity of the (006) reflection was notably higher. The magnet in Figure 3(bi), when subjected to a faster deformation rate, demonstrated a remanence value of 13.01 kG. Conversely, with a slower deformation rate, the magnet in Figure 3(bii) achieved a remanence value of 13.39 kG. This difference highlights the pronounced intensity of the (006) peak in Figure 3b, with the other peaks, such as (105), notably diminished.

**Figure 4:**
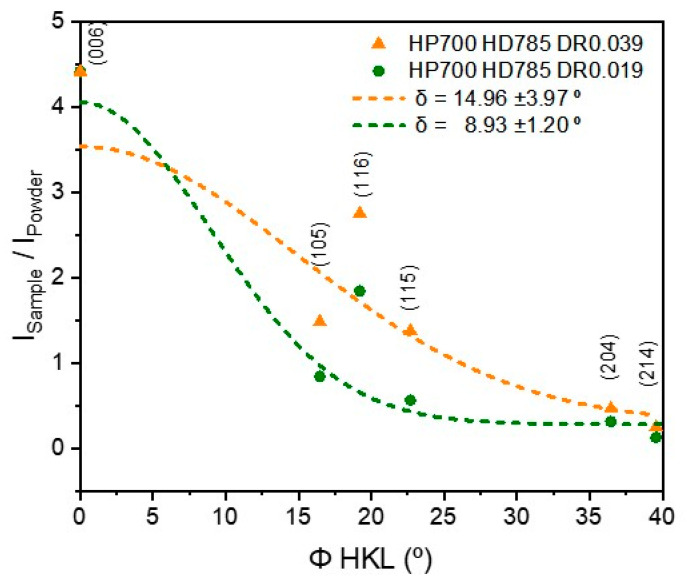
Half Gaussian fitted curves of orientation deviation δ (°) of hot-deformed samples produced by HP700 + HD785 with DR 0.019 and DR0.039.

To confirm the crystallographic alignment of the hot-deformed magnets for each process, a calculation model was employed, using the standard deviation of a Gaussian distribution for the relative intensity versus the angle between the normal of (HKL) and the c-axis of the Nd-Fe-B magnet (Figure 4) [29,30]. According to this model, the orientation deviation δ (expressed in (°)) can be related to the ratio of $I_{HKL}^{Sample}$ to $I_{HKL}^{Powder}$ as follows:

(1) $$\frac{I_{HKL}^{Sample}}{I_{HKL}^{Powder}}=A\cdot e^{\left(-\frac{\emptyset^{2}}{{2\delta }^{2}}\right)}$$

where *δ* (°) is the orientation deviation of Nd_2_Fe_14_B grains, ∅ (expressed in ∅ HKL (°)) is the angle between the normal of the (HKL) plane and the c-axis of the Nd-Fe-B sample, I is the intensity of the diffraction peaks of the samples ($I_{HKL}^{Sample}$) and that of the ideally isotropic powder ($I_{HKL}^{Powder}$) taken from the standard PDF card of the Nd_2_Fe_14_B phase (#39-0473), and A is a normalized parameter. A small value of σ indicates a strong (0 0 1) texture. The calculated orientation deviation values *δ* (°) are shown in Figure 4 and Table 2. In the fitted curves shown in Figure 4 for HP700 + HD785 with DR0.019, a decrease in the deformation rate from 0.039 to 0.019 mm/s led to a reduction in the orientation deviation from 14.96° to 8.93°.

The calculated orientation deviation is shown in Figure 4 and Table 2. As the hot-pressing temperature increased to 700 °C, these values decreased, suggesting a stronger (0 0 1) texture. A hot-pressing temperature of 700 °C, combined with the hot-deformation process temperatures of 785–815 °C and lower deformation rates, resulted in a reduced orientation deviation and enhanced remanence. The pressure at HP700 was believed to be more effective than that at HP675 for grain alignment. This is consistent with a study by Rong, who observed that decreasing the pressure loading rate can intensify texturing [31].

### 3.3. Optimization of NdFeB Magnet Properties: Response Surface Methodology Approach

The crystallographic alignment of hot-deformed magnets has been extensively debated. Although the definitive mechanism remains elusive, recent studies have explored elements that could aid in the deformation and alignment processes [32]. We employed response surface methodology combined with the BBD to investigate the key factors influencing this process. The use of the BBD in response surface methodology allows for the identification of optimal variable levels to obtain the desired response values. In particular, when considering three variables, it provides a method for determining the optimal experimental conditions with a minimal set of experiments. Table 3 presents the BBD matrix along with the responses for remanence (Response 1) and coercivity (Response 2), offering a detailed view of the experimental design and outcomes, thus laying the foundation for our analysis. This study identified three pivotal variables for the crystallographic alignment of hot-deformed NdFeB: the hot-pressing temperature (°C), hot-deforming temperature (°C), and deformation rate (mm/s) [18,33]. These factors are crucial for determining the optimal conditions for the crystallographic alignment.

We conducted a series of experiments and utilized the quadratic models represented by Equations (2) and (3) to elucidate the influence of the chosen factors on the crystallographic alignment, remanence, and coercivity of the hot-deformed magnets. Equations (2) and (3) model the influence of the processing parameters on the magnetic properties of the hot-deformed NdFeB magnets, where $T_{HP}$ represents the hot-pressing temperature, $T_{HD}$ represents the hot-deforming temperature, and $D_{DR}$ indicates the deformation rate. These equations were formulated based on extensive empirical data and refined through regression analysis to predict the changes in remanence Br and coercivity Hcj as functions of these parameters.

Equation (2) for Remanence (Br):

(2) $$Br=-591+1.854T_{HP}-0.132T_{HD}+542D_{DR}-0.001093T_{HP}^{2}+0.000254T_{HD}^{2}\;-0.000380T_{HP}T_{HD}-0.780T_{HP}D_{Dr}$$

This indicates how each processing parameter influences the magnet. The coefficients reflect the sensitivity of the remanence to changes in each parameter, highlighting the critical role of the deformation rate and the interactive effects between the pressing and deformation temperatures.

Similarly, Equation (3) for Coercivity (Hcj) is

(3) $$Hcj=03234+2.852T_{HP}+5.75T_{HD}-3064D_{DR}-0.001416T_{HP}^{2}-0.00312T_{HD}^{2}\;-5525D_{DR}^{2}-0.001167T_{HP}T_{HD}+2.65T_{HP}D_{DR}+1.97T_{HD}D_{DR}$$

This describes the dependence of the coercivity on the thermal and mechanical conditions during magnet processing. The negative coefficients of the deformation rate squared $D_{DR}^{2}$ suggest a significant decrease in coercivity with an increasing deformation rate, which is critical for optimizing magnet performance.

These formulations were supported by detailed statistical analyses, including analysis of variance (ANOVA), which confirmed the significant influence of $T_{HP}$ on both magnetic properties, particularly remanence, as demonstrated by the low *p*-value (0.001) for the $T_{HP}^{2}$ term.

**Figure 5:**
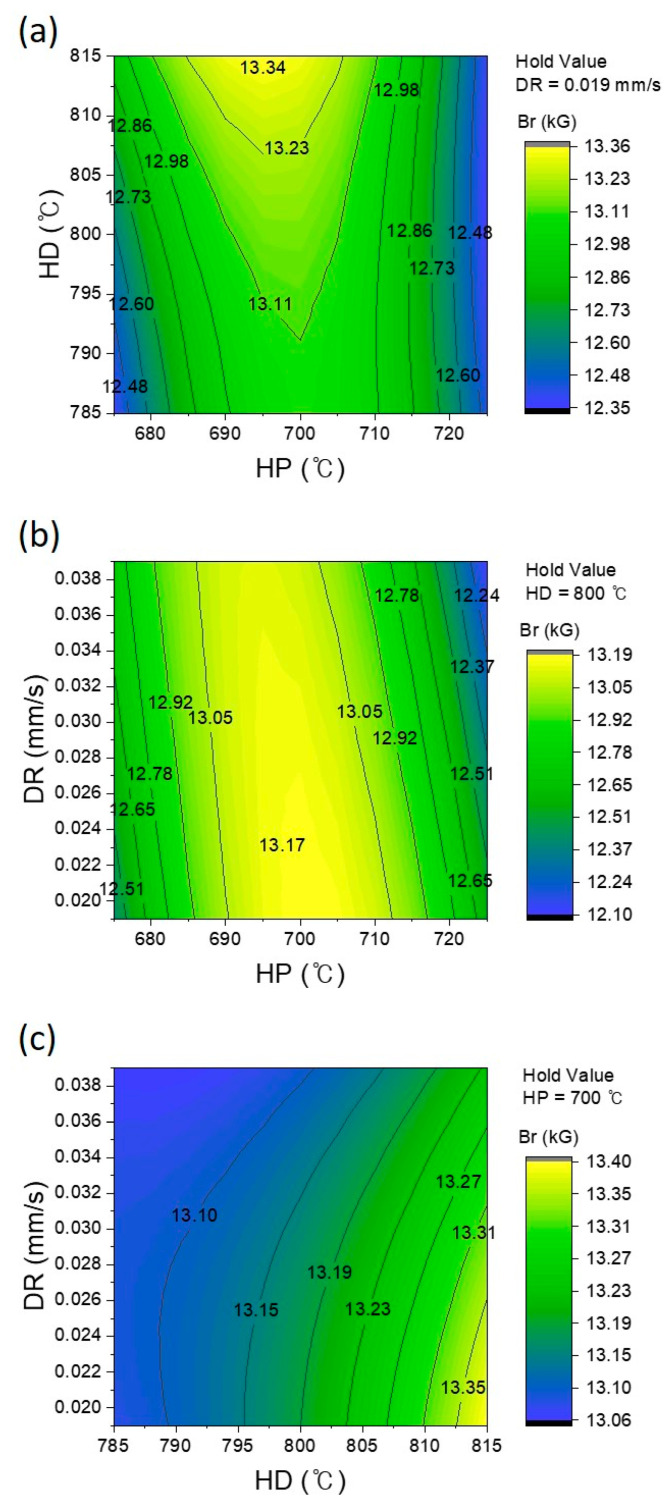
Contour plots for showing the remanence (Br) for (**a**) HP × HD at DR 0.019 mm/s, (**b**) HP × DR at HD 800 °C, and (**c**) HD × DR at HP 700 °C; HP represents the hot-pressing temperature, HD represents the hot-deforming temperature, and DR indicates the deformation rate.

The three-dimensional surface plots and contour plots, illustrated in Figure 5, provided further insights into the interaction effects of HP and HD at a constant DR. These plots demonstrated how variations in HP and HD, while maintaining a constant DR, affected remanence. From the ANOVA results in Table 4 and Figure 5c, we observed that maintaining the remanence value at 13.20 kG or above required a DR of 0.022 mm/s or lower, under conditions of HD800 or higher, while keeping the HP fixed at HP700.

**Figure 6:**
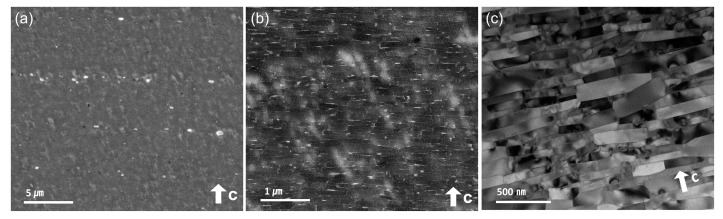
Microstructural features of the hot-deformed magnet processed under the conditions of HP700 + HD785 + DR0.019: (**a**,**b**) are BSE-SEM images, and (**c**) is a transmission electron microscope (TEM) image.

**Figure 7:**
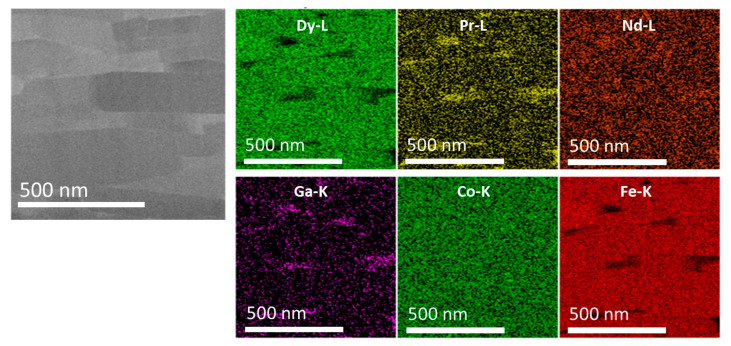
High-angle annular dark-field (HAADF) scanning transmission electron microscopy (HAADF-STEM) images and energy-dispersive X-ray spectroscopy (EDS) elemental maps for Dy, Pr, Nd, Ga, Co, and Fe in the hot-deformed magnet processed under the conditions of HP700 + HD785 + DR0.019.

Microstructure characterization using SEM and transmission electron microscopy (TEM), as shown in Figure 6, revealed a high remanence value of 13.39 kG in the HP700 + HD785 + DR0.019 hot-deformed sample. High-magnification images showed platelet-shaped grains neatly stacked together, with flat faces normal to the press direction. The platelet-shaped grains had an average width of 454.76 nm and an average thickness of 83.95 nm. The EDS mapping shown in Figure 7 confirmed the presence of Pr-L and Ga-K between the platelet-shaped grains. The role of these elements in promoting the crystallographic alignment through solution creep precipitation has been highlighted [34,35]. The optimal hot-pressing temperature of 700 °C played a pivotal role in achieving excellent crystallographic alignment in hot-deformed isotropic magnets with a uniform grain size and no defects or voids.

### 3.4. Impact of Stress Exponents and Deformation Conditions on the Magnetic Properties of the NdFeB Magnet

**Figure 8:**
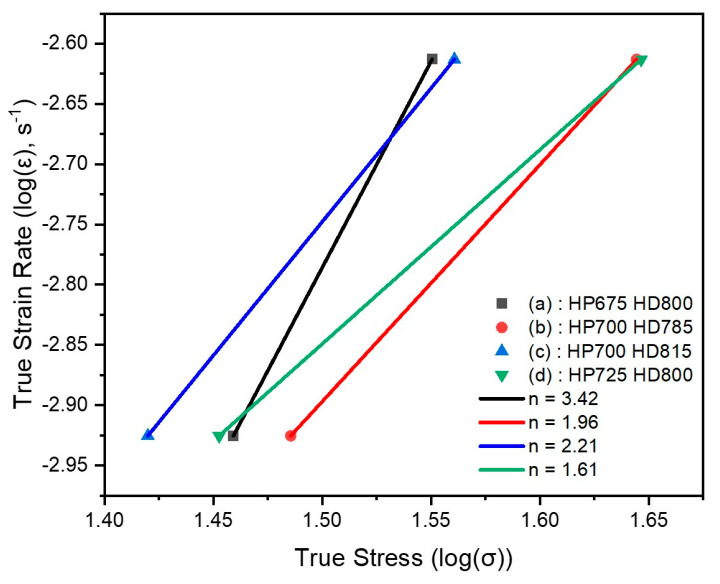
Logarithmic true strain rate versus logarithmic true stress curve, along with the stress exponent n in the BBD matrix for conditions (a):HP675 HD800, (b):HP700 HD785, (c):HP700 HD815, and (d):HP725 HD800.

Figure 8 illustrates the logarithmic relationship between the true strain rate and true stress for hot-deformed NdFeB, analyzed using the Dorn equation [36]. Through this analysis, we aim to examine how the conditions of hot deformation affect the grain alignment and remanence of the material. The data points represent different experimental conditions in the BBD matrix, each associated with a unique stress exponent n. At high temperatures, the relationship between the stress and strain rate can generally be expressed by the Dorn equation as follows:

(4) ε=A·σndm·e(−QRT)

Here, ϵ represents the strain rate, σ represents the stress, n represents the stress exponent, d represents the mean grain size, m represents the grain-size exponent, Q represents the activation energy, R represents the gas constant, and A represents a constant. Studies have identified different creep mechanisms with distinct stress exponents: Nabarro–Herring creep exhibits n = 1 and m = 2 [37]; Cobble creep also shows n = 1 and m = 3 [38]; grain boundary sliding with dislocations and superplasticity shows n = 2 [39,40]; subgrain creep or internal stress is characterized by n > 3 [41,42]. These values elucidate the relationship between the stress (σ) and strain rate (ϵ) under different deformation conditions, highlighting how each mechanism affects the response of the material to mechanical stress. Figure 8 illustrates this relationship, where the slopes of the graphs indicate the strain rate sensitivity, which is inversely proportional to the stress exponent, n.

In the HP675 + HD800 experiment, as depicted in Figure 8a, the true stress strain relationship shows that the calculated value of n is 3.42, which is consistent with other studies [41,42]. The HP700 + HD785 and HD815 experiments, shown in Figure 8b and c are consistent with Equation (4), with n having values of 1.96 and 2.21, respectively, corroborating prior studies [37,38]. The HP725 + HD800 experiment, shown in Figure 8d, also follows Equation (4), indicating a value of 1.61 for n [25,26].

In HP675 + HD800, n = 3.42; this value is typically associated with subgrain creep or internal stress. Such a high value of n indicates a significant stress concentration within the material or the activation of complex deformation mechanisms. Changes in the internal stress at high temperatures can play a significant role. In the HP700 + HD785 and HD815 experiments, n − 1.96 and n − 2.21, respectively. These values, being close to 2, suggest that the dominant deformation mechanisms involve grain boundary sliding and superplasticity. This indicates that deformation predominantly occurs between particles, and the microstructure is more responsive to strain. In such cases, deformation is sensitive to the particle size and the movement of boundaries. In the HP725 + HD800 experiment, n = 1.61; this value likely corresponds to diffusion-controlled mechanisms such as Nabarro–Herring creep or Cobble creep. The particle size significantly affects the creep rate. A lower n value suggests that the material is less sensitive to deformation.

**Figure 9:**
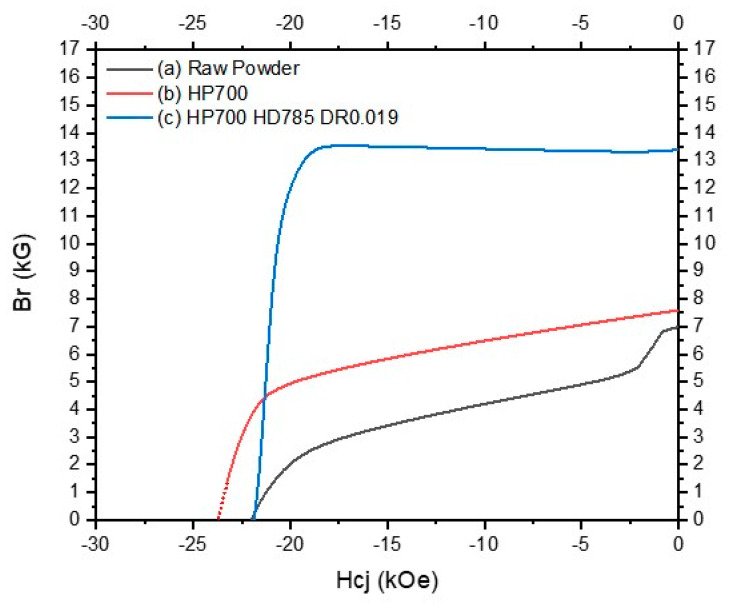
Demagnetization curves of hot-deformed magnets for (**a**) the raw magnetic powder, (**b**) after the hot-pressing process at 700 °C, and (**c**) after the hot-deformation process at 785 °C, with a deformation rate of 0.019 mm/s.

Note that in Figure 9, the demagnetization curves of the hot-deformed magnets show remanence values exceeding 13.2 kG, particularly those associated with the processes having stress exponent values of 1.96 and 2.21. Specifically, curve (c) in the figure, which represents the magnet processed at a hot deformation temperature of 785 °C and a deformation rate of 0.019 mm/s, achieved a significantly high remanence. A stress exponent close to 2, as demonstrated by curves (b) and (c), suggests that the deformation is sensitive to the particle size and boundary movement [39,40]. Thus, hot-pressed magnets manufactured at a hot-pressing temperature of 700 °C can induce movement at particle boundaries, which is critical for achieving the observed magnetic properties. Additionally, the MSDP showed significant plastic flow owing to the fine-grained microstructures, which are believed to have enabled the attainment of the high alignment of crystal grains and a remanence value of 13.39 kG. These observations, highlighted by evident distinctions in the demagnetization behavior across different stages shown in the figure, underscore the potential for achieving exceptional magnetic properties while accommodating complex material configurations.

To provide a clearer comparison and highlight the uniqueness and advantages of our study, we have summarized the demagnetization data along with other research in Table 5. This table helps in understanding the performance of our hot-deformed NdFeB magnets.

The data in Table 5 allow for a direct comparison between our study and other significant research in the field. Our sample, Nd_4.32_Pr_5.55_Dy_0.78_Fe_80.16_Ga_0.52_Co_4.47_B_4.19_, demonstrates a Br of 13.39 kG and a Hcj of 21.88 kOe, which are competitive with or superior to those reported in other studies. For instance, the study referenced in [43] reports a Br of 13.29 kG but a lower Hcj of 11.98 kOe. Similarly, the study in [44] achieves a high BHmax of 53 MGOe but employs a complex Dy_70_Cu_30_ press injection process.

By proposing a method that improves both remanence and coercivity without additional processing steps, our research highlights the efficiency and robustness of our hot-deformation magnet manufacturing process. Comparing our results with those of other studies underscores the effectiveness of our approach in producing high-quality NdFeB magnets.

## 4. Conclusions

We fabricated an optimized hot-deformed NdFeB magnet with good magnetic properties using the MSDP, which marked a significant advancement in the field. Through the application of the BBD matrix and response surface regression method, key processes and variables were considered and analyzed, with the hot-pressing temperature of 700 °C being identified as the most influential factor in determining the remanence value of the hot-deformed magnet.

Significant improvements in the crystallographic alignment were achieved by controlling the strain rate at 0.019 mm/s, leading to orientation deviation values below 10°. The resulting hot-deformed magnet exhibited remanence values of 13.4 kG and coercivity values exceeding 20 kOe, indicating excellent magnetic alignment. While this study focused primarily on the optimization of remanent magnetization owing to its critical influence on magnet performance, we acknowledge the importance of coercivity and its variability. A detailed analysis of coercivity, which exhibits a wide range from 21.11 to 23.65 kOe, is beyond the scope of this paper but will be comprehensively addressed in a forthcoming study. This subsequent analysis will allow us to delve deeper into the relationship among the coercivity, processing conditions, and microstructural characteristics of NdFeB magnets.

This study provides a novel understanding of creep deformation behavior in NdFeB hot deformation, highlighting the importance of considering the stress strain curve and the mechanisms of diffusion creep and grain boundary sliding. Furthermore, our efforts to scale-up magnet production using the MSDP demonstrated promising results. In the fabrication of scaled-up hot-deformed magnets in subsequent studies, we confirmed excellent magnetic properties with a remanence value of over 13.4 kG and a coercivity of over 23 kOe. This demonstrates the capability of the MSDP and response surface methodology of the BBD model to accurately predict the outcomes of larger-scale experiments, despite the low predicted R-squared value observed in the preliminary findings. We have limited our discussion to these outcomes.

In conclusion, the MSDP is promising for manufacturing near-net-shaped hot-deformed permanent magnets without compromising their magnetic properties. Future studies will focus on optimizing the MSDP and exploring its applicability to other compositions of permanent magnets, as well as conducting a thorough investigation into the relationship between the grain alignment and grain refinement and their influence on the magnetic properties.

## Figures and Tables

**Table 1:** Density (g/cm^3^), remanence (kG), and coercivity (kOe) corresponding to various hot-pressing temperatures (650 to 750 °C).

Hot Pressing Temperature (°C)	Density (g/cm^3^)	Remanence (kG)	Coercivity (kOe)
650	7.051	6.94	22.57
675	7.338	7.59	23.24
700	7.581	7.93	24.79
725	7.566	7.91	23.18
750	7.567	7.98	23.53

Theoretical Density: 7.59 g/cm^3^ (calculated based on the crystallographic parameters of Nd_2_Fe_14_B (Tetragonal crystal system).

**Table 2:** Remanence value (kG) and orientation deviation δ of the hot-deformed magnet corresponding to various hot-pressing temperatures (HP) (675 and 700 °C), hot-deformation temperatures (HD) (785, 800, and 815 °C), and deformation rates (DR) (0.019, 0.029, and 0.039 mm/s).

HP (°C)	HD (°C)	DR (mm/s)	Remanence (kG)	Orientation Deviation δ (°)
675	785	0.019	13.12	12.72
	800	0.029	12.16	18.58
	800	0.039	12.81	14.84
	815	0.029	12.34	12.25
700	815	0.019	13.45	5.75
	785	0.019	13.39	8.93
	785	0.039	13.01	14.95

**Table 3:** Box–Behnken design matrix with Response 1 (remanence) and Response 2 (coercivity).

Standard Order	Run	Factor X_1_: T_HP_ (°C)	Factor X_2_: T T_HD_ (°C)	Factor X_3_: D_DR_ (mm/s)	Response 1: Br (kG)	Response 2: Hcj (kOe)
10	1	700	815	0.019	13.45	21.11
2	2	725	785	0.029	12.21	22.94
7	3	675	800	0.039	12.81	21.28
11	4	700	785	0.039	13.01	23.07
14	5	700	800	0.029	13.07	23.65
1	6	675	785	0.029	12.34	21.24
6	7	725	800	0.019	12.49	21.80
15	8	700	800	0.029	13.27	23.44
13	9	700	800	0.029	13.15	23.64
5	10	675	800	0.019	12.15	22.62
3	11	675	815	0.029	13.12	22.04
9	12	700	785	0.019	13.39	21.88
8	13	725	800	0.039	12.37	23.11
4	14	725	815	0.029	12.42	21.99
12	15	700	815	0.039	12.93	23.48

**Table 4:** Analysis of Variance (ANOVA) for the response surface methodology and fit statistics of models.

Analysis of Variance (ANOVA)					
Source	DF	Adj SS	Adj MS	F-Value	*p*-Value
Model	7	2.27616	0.32517	4.75	0.029
Linear	3	0.24192	0.08064	1.18	0.385
_HP_	1	0.10811	0.10811	1.58	0.249
T_HD_	1	0.11761	0.11761	1.72	0.232
D_DR_	1	0.01620	0.01620	0.24	0.642
2FI2	2	1.80091	0.90046	13.14	0.004
T^2^_HP_	1	1.76345	1.76345	25.73	0.001
T^2^_HD_	1	0.00965	0.00965	0.14	0.719
Quadratic	2	0.23333	0.11666	1.70	0.250
T_HP_∙T_HD_	1	0.08123	0.08123	1.19	0.312
T_HP_∙D_DR_	1	0.15210	0.15210	2.22	0.180
Residual	7	0.47968	0.06853		
Lack of fit	5	0.45941	0.009188	9.07	0.102
Pure error	2	0.02027	0.01013		
Total	14	2.75584			
Fit statistics of models					
S	0.258975				
R^2^	82.90%				
Adjusted R^2^	65.80%				
Predicted R^2^	0.00%				

**Table 5:** Comparison of demagnetization data with other hot deformed magnet studies.

Sample	Br (kG)	Hcj (kOe)	BHmax (MGOe)	Reference	Additional Notes
Nd_4.32_Pr_5.55_Dy_0.78_Fe_80.16_Ga_0.52_Co_4.47_B_4.19_	13.39	21.88	44.78	This study	HP700, HD785, DR0.019
Nd_13.6_Fe_73.6_Ga_0.6_Co_6.6_B_5.6_	13.29	11.98	43.3	[43]	High-stress low-temperature rapid deformation.
Nd_13.6_Fe_73.6_Ga_0.6_Co_6.6_B_5.6_	14.26	13.81	49	[23]	Slow plastic deformation at low temperatures
Nd_9.46_Pr_3.06_Fe_77.26_Co_3.82_Ga_0.46_B_5.95_ + Tb_20_Dy_10_Nd_40_Cu_30_ and Nd_80_Cu_20_	12.9	24.3		[17]	Two-step eutectic GBD
Nd_13.79_Pr_0.1_Fe_75.39_Co_4.57_Ga_0.43_B_5.77_ + Dy_70_Cu_30_	14.9	9.7	53	[44]	Dy_70_Cu_30_ Press injection

## Data Availability

The data presented in this research are available on request from the corresponding authors.
